# Facilitating Machine Learning‐Guided Protein Engineering with Smart Library Design and Massively Parallel Assays

**DOI:** 10.1002/ggn2.202100038

**Published:** 2021-12-07

**Authors:** Hoi Yee Chu, Alan S. L. Wong

**Affiliations:** ^1^ Laboratory of Combinatorial Genetics and Synthetic Biology School of Biomedical Sciences The University of Hong Kong Hong Kong 852 China; ^2^ Electrical and Electronic Engineering The University of Hong Kong Pokfulam Hong Kong 852 China

**Keywords:** combinatorial mutagenesis, machine learning, protein engineering, infologs, functionality prediction

## Abstract

Protein design plays an important role in recent medical advances from antibody therapy to vaccine design. Typically, exhaustive mutational screens or directed evolution experiments are used for the identification of the best design or for improvements to the wild‐type variant. Even with a high‐throughput screening on pooled libraries and Next‐Generation Sequencing to boost the scale of read‐outs, surveying all the variants with combinatorial mutations for their empirical fitness scores is still of magnitudes beyond the capacity of existing experimental settings. To tackle this challenge, in‐silico approaches using machine learning to predict the fitness of novel variants based on a subset of empirical measurements are now employed. These machine learning models turn out to be useful in many cases, with the premise that the experimentally determined fitness scores and the amino‐acid descriptors of the models are informative. The machine learning models can guide the search for the highest fitness variants, resolve complex epistatic relationships, and highlight bio‐physical rules for protein folding. Using machine learning‐guided approaches, researchers can build more focused libraries, thus relieving themselves from labor‐intensive screens and fast‐tracking the optimization process. Here, we describe the current advances in massive‐scale variant screens, and how machine learning and mutagenesis strategies can be integrated to accelerate protein engineering. More specifically, we examine strategies to make screens more economical, informative, and effective in discovery of useful variants.

## Graphical abstract

Integrating protein design and variant screening with machine learning facilitates the discovery of useful variants for applications including antibody therapy and vaccine generation. This perspective describes how the prioritization of variant testing maximizes the power of machine learning for protein engineering, strategies for designing a smart screening library, and considerations for integrating massively parallel assays with machine learning.

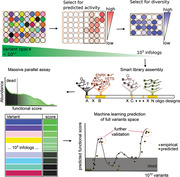

## 1 Introduction: A Case for Machine Learning‐Guided Assay

With the explosion of DNA sequencing data, identification of variants in the genome within and across species from comparative studies or genome profiling has become commonplace in biology. Variations raise questions in evolution (studying how mutations arise and sustain), pathology (characterizing variant effects in diseases), and functionality (defining mutational effects on protein folding, dynamics, and interactions). While studying naturally occurring variants provides valuable knowledge in diseases and biodiversity, rational design and directed evolution are employed to optimize or re‐purpose proteins for human use. Biocatalysts and protein‐based drugs are of great interest in industries; there is an ongoing pursuit for the improved efficiency, versatility, and specificity of these proteins.

**Figure 1:**
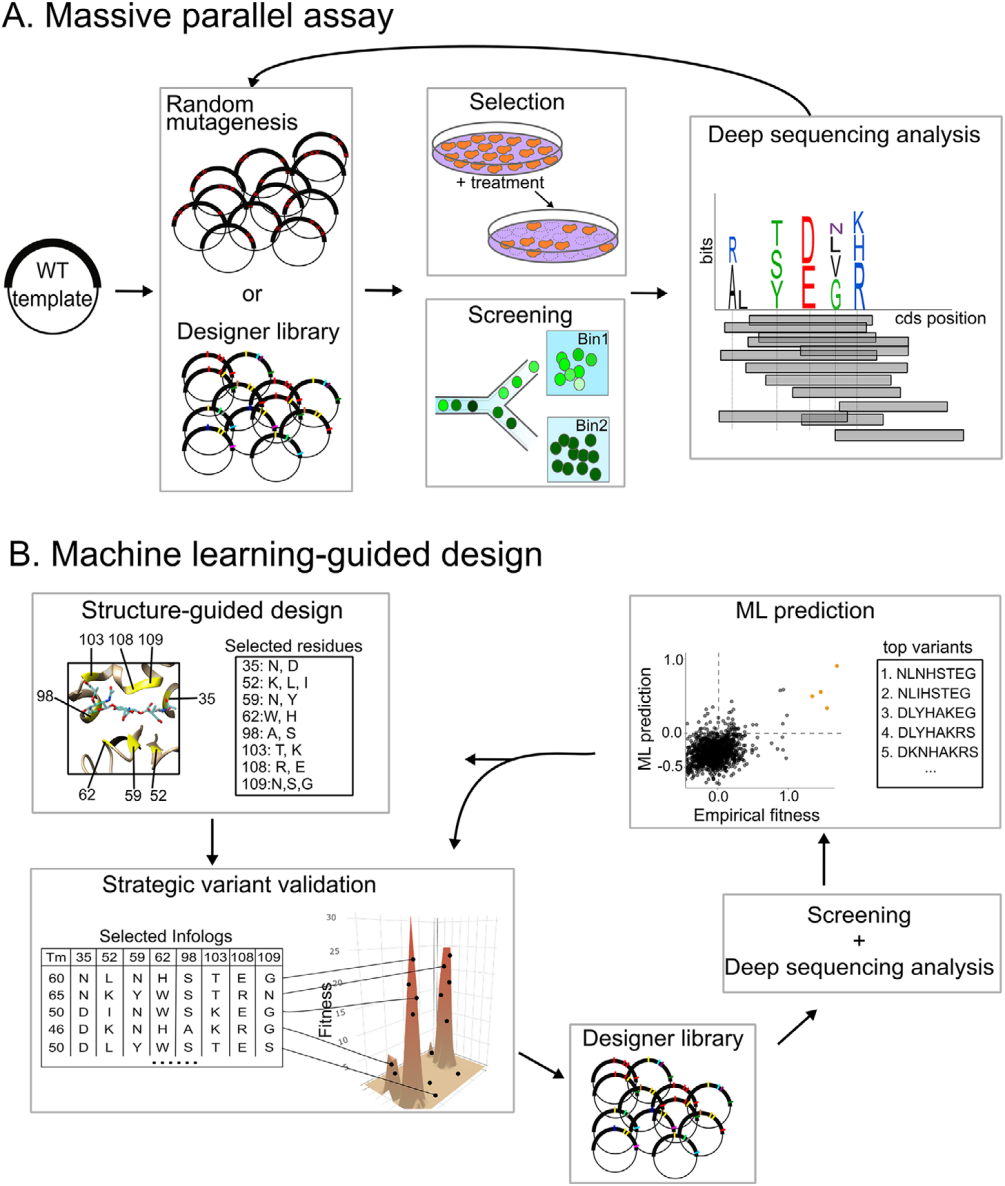
Comparing typical massively parallel assays to machine learning‐guided screens. A) Workflow of an exhaustive screen for top variants. Starting with the wild‐type (WT) template, mutations are introduced via random mutagenesis, oligo synthesis, or CRISPR‐based editing in the designer library. The plasmids containing different variant sequences are transformed or transfected into the host cells. Positive selection or FACS‐based screen is performed to isolate functional variants. The coding sequences of the variants are retrieved via deep sequencing. Further analyses may reveal sites that are tolerant to mutations and the preferred residues of the site, presented in the logo plot for visualization. These top variants are isolated and then seed a new round of library construction for further optimization. B) Workflow of an ML‐assisted screen. First, important amino acid sites are identified, for example from the crystal structure. By rational structure‐guided design, a restricted set of residues are selected for each site subjected to engineering. Given that the fitness landscape is unknown a prior to the researcher, a small sub‐sample of variants can be selected based on stability (i.e., melting temperature (*T_m_*)) or maximized mutual information. Then, these selected variants are constructed in the designer library and experimentally validated via a high‐throughput screen and deep sequencing. Such a subset of empirical fitness measurements is used for training the ML algorithm. The accurate ML model is then used to predict fitness values for the entire library and select the top variants for further validation. Alternatively, the top variants can then undergo another round of rational design for further augmentation.

To identify a desirable variant among the many non‐functional counterparts, effective screening systems are built to survey the “trait” of 10^4^–10^12^ variants in a pooled manner.^[^ ^1^, ^2^, ^3^, ^4^, ^5^, ^6^ ^]^ The fundamental components of these screening systems are 1) library construction, 2) functional screen, and 3) selection of the “better” variants (**Figure** **1**A). Such three‐step process may be iterated for several cycles until the desirable variants are successfully identified and subjected to more vigorous evaluations downstream. The key limit of these screens (i.e., the number of variants that could be screened experimentally), depends on the number of sites and mutations included in the library and the throughput and accuracy of the selection.

Incorporation of computational methods into these screening platforms makes in‐silico screening possible, thus further accelerates the mining of useful variants.^[^ ^7^ ^]^ Recently, ML‐mediated in‐silico screens allow researchers to search libraries of thousands of variants for top‐performing candidates using fitness information from no more than 100 experimentally validated variants.^[^ ^8^, ^9^, ^10^ ^]^ In one example, Mason et al. performed an in‐silico screen of 10^8^ trastuzumab variants for their enhanced HER2 binding specificity based on 10^4^ data points from a deep mutational scan in mammalian hybridoma cells.^[^ ^11^ ^]^ This study demonstrates the immense potential of machine learning (ML) to facilitate protein engineering by reducing the workload of variant profiling in cell lines, thus providing more realistic screening results compared to in‐vitro settings. Additional to optimization, ML‐assisted screens achieve great successes in protein diversifications,^[^ ^12^, ^13^, ^14^, ^15^ ^]^ rivaling the data‐driven or Rosetta‐based *de novo* protein design methodologies.^[^ ^16^, ^17^, ^18^, ^19^, ^20^ ^]^ ML‐based generations of new antibody sequences can produce diverse binders to specific antigens;^[^ ^14^ ^]^ such generative framework can also create a new generation of antibody libraries with enhanced expression and developability. The novel sequences can be used for the next round of in‐silico screening or as training data to improve the model performance.^[^ ^21^ ^]^

ML enables probing the extremely large space of variants with a smaller but informative subset of variants examined empirically. One of the main challenges in harnessing the power of ML is to generate a “useful dataset” that maximizes predictive accuracy. A strategy to accurately extrapolate the very few experimentally validated variants to infer fitness of the complete landscapes involves both a smart sampling strategy to capture some “positives” among these experimentally validated variants and an accurate ML model.

Below, we provide our perspectives on machine learning‐guided massive parallel assays; we summarize the prioritization of variant testing under the workflow of ML‐assisted protein engineering and discuss how “smart” library design and massive parallel assays complement the ML model with useful training datasets.

## 2 A Brief Summary of Machine Learning‐Assisted Protein Engineering

Numerous reviews and articles about ML on protein engineering already document its application and workflow,^[^ ^7^, ^22^, ^23^, ^24^, ^25^, ^26^ ^]^ highlighting the importance of both amino‐acid representations and ML algorithms in generating high‐accuracy models.

Amino acid representations contain additional features augmenting the sequence‐based embedding for ML. There are sequence‐based pretrained representations derived from NLP models,^[^ ^27^, ^28^, ^29^, ^30^, ^31^, ^32^ ^]^ structural descriptors,^[^ ^33^, ^34^, ^35^, ^36^ ^]^ and descriptors that capture physio‐biochemical properties of amino acids.^[^ ^37^, ^38^ ^]^ Instead of complex metrics derived from protein structure prediction software, more intuitively explanatory descriptors such as the number of contacts to other amino acid residues are excellent predictors.^[^ ^39^, ^40^, ^41^ ^]^ Evolutionary descriptors including conservation score and PSSM weight are proven to be also extremely useful in predicting mutational effect^[^ ^40^, ^42^, ^43^ ^]^ and site‐wise tolerance to mutations. The use of multiple sequence alignments (MSA) to generate useful embeddings from transformer language models is under active research.^[^ ^9^, ^31^, ^44^, ^45^ ^]^ Apart from producing accurate 3D structure models,^[^ ^46^ ^]^ language models built from MSA can perform zero‐shot fitness inference on proteins with abundant homologous sequence information.^[^ ^47^ ^]^ Among the many ML algorithms, neural networks,^[^ ^37^ ^]^ tree‐based models,^[^ ^33^, ^38^ ^]^ and Gaussian processes^[^ ^8^, ^48^, ^49^ ^]^ have been commonly adopted for variant fitness inference and show high accuracy in selecting functional variants. Applications of generative models to extract useful features from amino acid sequences or 3D structural attributes and generate optimized protein sequences are gaining popularity,^[^ ^12^, ^14^, ^21^, ^25^, ^50^ ^]^ because of the models’ capability in generating synthetic sequences that can serve as future training data^[^ ^21^ ^]^ as well as novel designs.^[^ ^14^ ^]^

## 3 Sampling Strategies to Maximize Machine Learning Power

Evaluating the combined effects of beneficial mutations is the key to protein optimization. However, synthetic combinatorial libraries usually generated a large portion of non‐functional variants due to the shape of the fitness landscape,^[^ ^51^, ^52^ ^]^ substantial epistasis interactions in play,^[^ ^53^, ^54^ ^]^ and the nature of the synthetic library (i.e., low expression, host toxicity, etc.).^[^ ^55^ ^]^

Scarcity of functional variants in the library creates a class representation bias that makes ML training difficult.^[^ ^56^ ^]^ For example, only 2.4% of high fitness variants are present in the GB1 fully saturated mutagenesis library that is used as a benchmark of many ML packages of modeling fitness effects.^[^ ^56^ ^]^ Another test case is redirecting a zinc deaminase to catalyze organophosphate hydrolysis;^[^ ^57^ ^]^ mutants possessing the quadruple mutations (D19S, F61T, A183I and D296A) necessary for endowing such new reaction are extremely rare in a saturation mutagenesis library spanning 12 amino‐acid sites.

One solution is to deliberately enrich training data with variants possessing non‐zero fitness scores.^[^ ^56^ ^]^ Such educated guesses were carried out via selecting variants with high stability based on Rosetta energy scores. Alternatively, one can sample representative variants that maximize sequence diversity.^[^ ^48^, ^51^, ^58^ ^]^ Indeed, that is the concept behind the design of the experiment (DOE) method that systematically generates a small set of gene variant “infologs” to minimize co‐variations between amino acid substitutions and ensure uniform sampling (Figure 1B). ML on these information‐rich infologs accurately confers combinatorial mutation effects on glutathione transferases detoxification.^[^ ^51^, ^58^ ^]^

Adaptive sampling^[^ ^59^, ^60^ ^]^ provides another solution in directing experimental efforts to informative variants. The uncertainty sampling‐based active learning approach focuses on the high‐fitness, high‐uncertainty candidates in each iteration of the ML training.^[^ ^8^, ^60^ ^]^ Briefly, iterations of ML are trained on the empirical data of the most uncertain candidates indicated in the ML model. After each round of ML training, new candidates with high uncertainty are identified and selected for experimental validation to update the ML model (Figure 1B).

Although active learning only outperforms randomized input for ML when the training data size exceeds 3,000, this sample size still only accounted for 17% of the entire dataset for chemical reactions.^[^ ^61^ ^]^ Using this adaptive sampling approach, Greenhalgh et al.^[^ ^10^ ^]^ performed ten rounds of design‐test‐learn to optimize fatty alcohol production of ACP reductase in vivo, where experimentally measuring fatty alcohol titer for the individual synthetic variant is expensive. Briefly, the authors began with surveying 20 representative variants from the combinatorial library of 4374 sequences and trained a Gaussian process (GP) sequence‐function model to identify top‐performing variants. In each round, 5–12 variants designed based on the upper confidence bound criterion were experimentally validated and added to the model as training data. Finally, after 10 rounds of ML and experimental validation of a total of 96 variants, four top‐performing chimera fatty acyl reductases were identified. This study again demonstrates that iterating between ML and experimental validation is an extremely effective approach in protein optimization.

We next explore how these sampling strategies that facilitate ML predictions on protein functions can be implemented experimentally via smart library design and massive parallel assay.

## 4 Strategies for Designing a Smart Screening Library for Machine Learning

**Figure 2:**
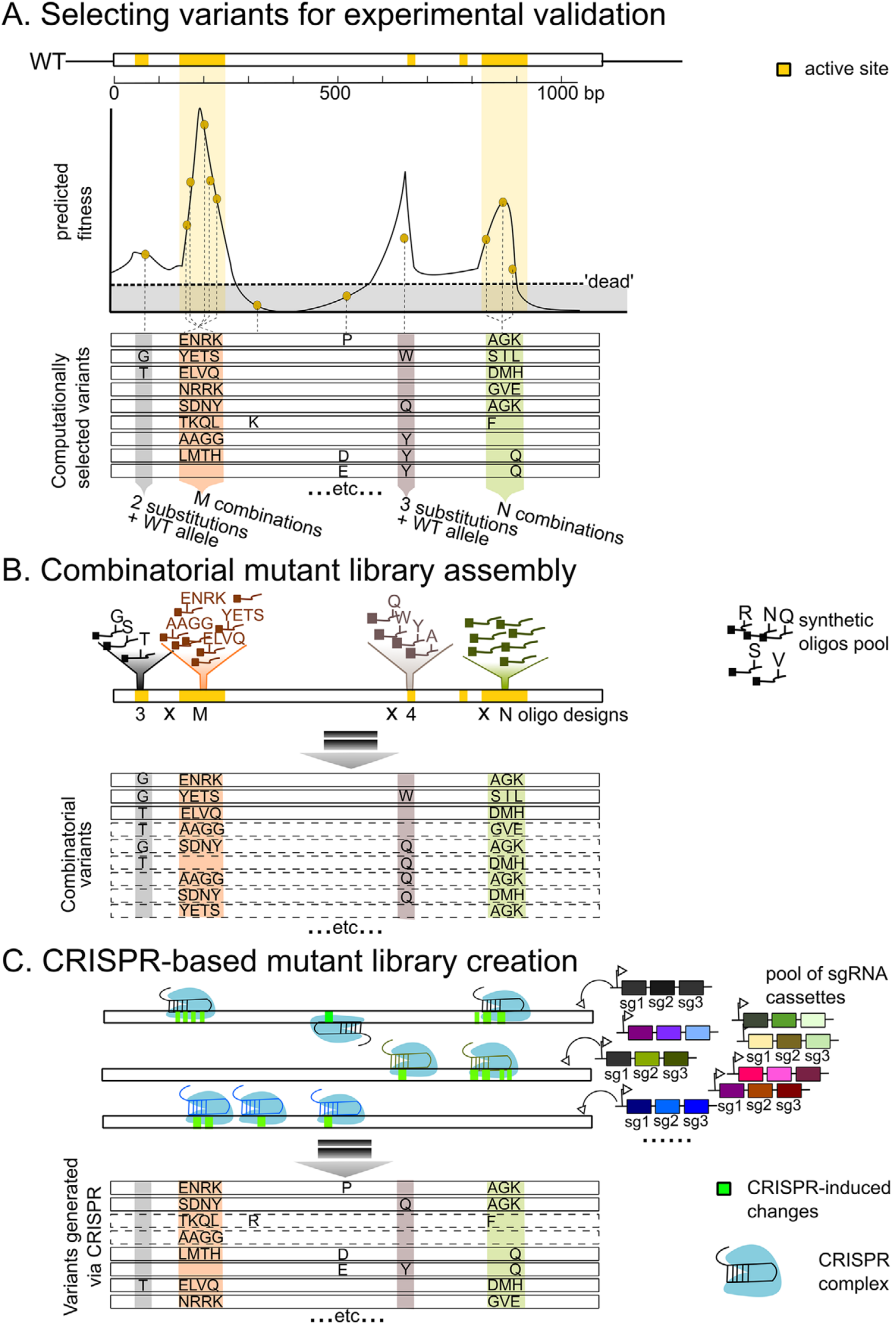
Smart library construction that assists machine learning‐guided protein engineering. A) Schematic diagram showing how variants are selected for experimental validation. In this example, the wild‐type (WT) protein‐coding sequence spans over 1 kilobase and contains five regions (highlighted yellow) forming the active site. With some preliminary data (i.e., from the deep mutational scan) or structure‐guided modeling, the fitness of the variants is predicted. Here, beneficial mutations leading to high fitness variants are clustered in four regions of the protein: two substitutions and WT allele (grey zone); combinations of four consecutive substitutions (orange zone); 3 substitutions and WT allele (dark‐brown zone); and combinations of three consecutive substitutions (avocado zone). To downsize the number of variants validated experimentally, a subset of the variants is selected computationally so that 1) plenty of “non‐dead” variants are included, and 2) sequence diversity is high. It is not feasible to generate these variants using random mutagenesis or site‐saturated mutagenesis. Instead, other strategies such as combinatorial library assembly and CRISPR‐based approach are needed to build such designer library. B) Combinatorial mutant library assembly. One solution is to focus on the regions that some of the mutation combinations may confer high fitness. For each region, pools of synthetic oligos corresponding to the specific mutation combinations are cloned into the WT template. A final mix of all mutational combinations across the four regions is generated using strategies such as CombiSEAL^[^ ^75^ ^]^ or other seamless ligation methods. The computational selected variants harboring correct mutational combinations (variants in solid lines) constitute a portion of the entire library; combinatorial variants that were not selected for experimental validation are also present (variants in dash lines), since it is difficult to control precisely which oligos are cloned into each fragment. C) CRISPR‐based approach to introduce precise mutations per variant. Specific sgRNA cassettes are employed to program CRISPR editing on the WT template. The sgRNA cassette directs the CRISPR complex to introduce changes (highlighted in green) in specific genomic regions. Using the specific, high‐order combinatorial sgRNA cassettes,^[^ ^82^ ^]^ a set of desired variants can be generated. The computationally selected variants should in theory constitute most of the library. Nonetheless, due to CRISPR off‐target effects and inactive guides, a portion of variants (in dashed lines) constructed may have no or incorrect mutations. Also, the activity of CRISPR‐based base editors will need a further boost to enhance its editing efficiency.

Careful design of the library is crucial for ML or statistical inference on the variant effect (**Figure** **2**A). When a Next‐Generation Sequencing (NGS)‐based survey is used, the inaccuracy of the variants’ fitness increases with the number of variants screened.^[^ ^62^ ^]^ Increasing the number of replicates and NGS read depth is necessary to compensate for the massive library size. In addition, the experimental efforts in generating the variants should be factored in. Together, library construction becomes the first key element in creating accurate ML models to describe the variant's fitness.

### 4.1 Deep Mutational Scans to Survey Mutational Effects

Random mutagenesis employed in deep mutational scans (DMS) is widely used in directed evolution and massive‐parallel assays. Hypermutation^[^ ^63^, ^64^ ^]^ and continuous evolution^[^ ^65^ ^]^ methods such as MAGE^[^ ^66^ ^]^ and PACE^[^ ^67^ ^]^ further increase the number of amino acid‐changes to 10–20 mutations in a variant by boosting the host genome mutation rate, accelerating the search for high‐performance variants.

Mutation effect estimations from DMS experiments reveal structurally and functionally important sites.^[^ ^68^, ^69^, ^70^, ^71^ ^]^ Nonetheless, DMS does not generate informative variants for protein repurposing, especially when the new sought‐after function does not align with the natural function of the protein since the current active site no longer fits the new substrate.^[^ ^72^ ^]^ Instead, focused mutagenesis and rational design are better suited to redesigning the active site within the current protein scaffold once the protein structure is available or active site residues are identified.

### 4.2 Designer Libraries Customized for Machine Learning In‐Silico Screen

To identify the best combinations of substitutions at a few key sites that dominate binding or enzymatic activities, saturation mutagenesis is an effective strategy to employ, generating all amino acid substitutions at a handful of amino acid sites via degenerate codons. However, the library size exponentially increases at a rate of 20*^N^* as the number of sites subjected to saturation mutagenesis increases. To limit the library size and reduce the screening effort, one may resort to structure‐guided design, restricting the number of substitutions screened per site and building a rationally designed library with fewer combinations.

Combinatorial libraries targeting multiple sites with a few shortlisted amino acid residues per site can be constructed through cloning with designer DNA oligos or trinucleotides (Figure 2B). With gene synthesis and homology‐based cloning strategies, structure‐guided SCHEMA recombination libraries that generate chimeras from blocks of parental homologous sequences can introduce over 70 mutations per chimera from its closest parent.^[^ ^73^ ^]^ Another important feature of targeted library design is the linking of genotypes to a molecular barcodes to accommodate short‐read Illumina sequencing. For instance, DropSynth 2.0 ^[^ ^74^ ^]^ allows the construction and barcoding of 1500 specific variants from hybridizing microarray‐derived oligos in droplets. Furthermore, Choi et al. demonstrated that CombiSEAL^[^ ^75^ ^]^ can survey multiple mutants possessing mutations hundreds of base pairs apart using a smart design of library cloning and barcoding scheme. Combinatorial mutagenesis generates and barcodes the full combination space of selected substitutions with certain extent of redundancy, since the information of lower‐order mutations (single and double mutants) is enclosed in higher‐order mutations (multi‐mutants).^[^ ^76^ ^]^ How to incorporate the DOE method in large‐scale combinatorial libraries to generate specific infologs remains to be addressed.

Departing from the combinatorial construction methods, the CRISPR‐based method is the emerging theme as it generates variants with precisely defined mutations (Figure 2C). CRISPR‐directed mutagenesis has proven to be extremely useful in generating specific disease variants for endogenous loci.^[^ ^77^, ^78^, ^79^ ^]^ Meanwhile, diverse antibody complementarity determining region 3 (CDRH3) was cloned via CRISPR mediated homology‐directed mutagenesis, achieving a library size of >10^5^ in mammalian hybridoma cells.^[^ ^11^ ^]^ Introducing precise mutations across multiple sites is made possible by a combinatorial CRISPR screen that utilizes multiple‐sgRNAs cassettes.^[^ ^80^, ^81^, ^82^ ^]^ CRISPR‐based methods, albeit suffering from some off‐target effects, still possess higher precision compared to MAGE (350 mutations after 20–35 evolution cycles)^[^ ^83^ ^]^ and synthetic oligo assembly such as DropSynth (≈20% success assembly rate). Fine‐tuning the CRISPR editing consequences^[^ ^84^ ^]^ will be extremely beneficial for generating precise variants in future projects.

As discussed in the previous section, a smart design library boosts ML predictive power by focusing surveying efforts on information‐rich variants. Oligo synthesis and CRISPR are useful tools in building massive, pooled libraries of variants with a set of focused substitutions. With the increase in the sequence length and scale of oligo synthesis and continuous improvement in CRISPR technology, we can foresee that more precise library construction is achievable in the future to generate computationally designed or selected variants en masse. Now, we turn to the screening system that is essential to generate functional fitness scores for ML.

## 5 Considerations on Integrating Massively Parallel Screening Platforms with Machine Learning

Screening systems perform the important role to tie the genotype to its phenotypic performance in a given environment. The most common approach is selection on cell survival or fluorescence‐activated cell sorting (FACS)‐based screens are followed by deep sequencing that identifies the “winning” variants by their enrichment in the filtered bin or selection endpoint. Thus far, massively parallel assays are almost built‐in with ML predictions on phenotypic values.^[^ ^11^, ^15^, ^39^, ^49^, ^56^, ^85^, ^86^, ^87^, ^88^ ^]^ While massive parallel assays are fast and cost‐efficient in evaluating multi‐mutation effects, mitigation of experimental noise remains an important task to make phenotypic scoring accurate and reproducible.^[^ ^71^, ^89^ ^]^ Gelman et al.^[^ ^90^ ^]^ proposed several useful recommendations on how to create reproducible multiplexed functional assays (MAVEs) useful for studying clinical variants; here we discuss some of them that are also applicable in ML‐assisted in‐silico screen.

### 5.1 Machine Learning‐Compatible Fitness Scoring

First, the assay should distinguish meaningful differences. This is ideal to separate functional variants from their non‐functional counterparts. Hereinto, selection on cell survival would be an excellent reporter. Similarly, competitive growth assays are highly effective in identifying variants conferring growth advantages over a short time frame. Although survival/growth‐based selection can handle a massive library of up to 10^8^ mutants,^[^ ^91^ ^]^ building a reporter system that can convert protein functionality to growth can be challenging. Besides, ML prediction quality improves when positive selections with lower stress were conducted, as a broader range of functional scores were collected and more variants were surveyed.^[^ ^39^ ^]^ Together, a reporter system independent of cell survival appears to be more beneficial for ML‐guided screens.

Imaging‐based screen is a useful solution to assess functional phenotypes delineated from survivals, where the phenotypic readout in fluorescence level enables sorting, morphological and genotype profiling.^[^ ^92^ ^]^ Although most of the current screens rely on FACS to isolate “winning” variants, future screens may shift to high‐throughput imaging combined with computer vision,^[^ ^93^ ^]^ especially in screens for protein‐based drugs that both drug potency and toxicity can be captured in real‐time through observing the morphology of treated‐cells under high‐resolution microscopes.

### 5.2 Screens of Biological Relevance

Second, the assay design should provide the relevant environment for functionality assessment. The choice of screening/selection methods combined with the host determines the number of variants that can be assessed in one experiment as well as the usefulness of the screen. Balancing the trade‐off between the screening capacity and the selection environment should be factored into the choice of reporter assay.

For example, the merits of using mRNA display to screen up to 10^13^ variants may be diminished for proteins that undergo extensive post‐translational modifications; yeast display (screening up to 10^9^ variants) could be used as an alternative.^[^ ^94^ ^]^ Further, yeast complementation assays on human proteins are particularly useful to study variants of clinical importance.^[^ ^95^ ^]^

As a better disease model, pooled multiplexed CRISPR screens are performed in human cell lines with specific disease characteristics to identify useful drug targets.^[^ ^96^, ^97^ ^]^ However, CRISPR‐based modifications are greatly constrained by the multiplicity of infection (MOI) and FACS throughput; such setup ceilings at about 10^5^ variants.^[^ ^98^ ^]^ Recent integration of single‐cell sequencing that can evaluate many different phenotypes greatly increases both throughput and resolution.^[^ ^98^, ^99^, ^100^ ^]^

### 5.3 Screens with High Reproducibility

The last but also one of the most important points is about quality control of the screen to ensure reproducibility. Using HSP90 as an example, DMS data deposited in MaveDB^[^ ^101^ ^]^ from multiple labs shares high correlations in functional scores (*R* ^2^ = 0.4038),^[^ ^102^ ^]^ exhibiting high reliability. Experiments under similar conditions clustering together in a PCA plot is another reassuring sign for results reliability.

One obvious measure to boost reproducibility is producing multiple biological replicates for each screen that share a high correlation in the functional scores and having the scores denoised with Enrich2,^[^ ^103^ ^]^ PACT,^[^ ^40^ ^]^ and MAVE‐NN.^[^ ^104^ ^]^ Alternatively, tagging multiple barcodes to a variant in a single experiment can facilitate reliable assessment. Although this method is adopted in MAVEs of enhancers, it is not common in protein variant assessments. An alternative approach is to introduce multiple synonymous variants for each protein phenotype.

It is also important for an expression‐based enrichment assay to start with a balanced library with the same starting frequency for each mutant. Unevenly pooled library introduces high variance in enrichment levels after selection,^[^ ^62^ ^]^ and software like MAUDE^[^ ^105^ ^]^ is dedicated for correcting such errors. To reduce technical noise, controlling per cell plasmid numbers using the Equalizers plasmid^[^ ^106^ ^]^ can make expression‐based assays more robust.

In sum, the throughput of the screen, experimental setup, and accuracy of the reporter gene are key components for generating good input functional scores for ML.

## 6 Concluding Remarks and Outlook

An ML‐guided approach is necessary to transverse the sequence‐function space effectively in the search of useful protein variants. Incorporation of ML greatly reduces the experimental burden in an exhaustive screen; accurate approximation of all the variants' fitness is achieved using merely 15% or fewer empirical measurements. Nonetheless, the potential of ML is only fully realized when provided with informative variant fitness scores and descriptive features. Generating a set of useful training data for ML protein design requires strategic library design to capture “informative” variants possessing diverse mutation combinations followed by a massively parallel assay that measures the functional score reliably.

However, library design and screening platforms are overlooked or only investigated retrospectively thus far in ML‐focused studies. An important next step is to factor in the ML component in both library construction and high‐throughput screen in future projects, so that we can maximize the utility of ML in the search of useful variants. Most importantly, the paradigm of using random mutagenesis for library construction can be replaced with methods that introduce substitution concisely such as combinatorial mutagenesis and CRISPR‐based methods to accommodate the downstream sequencing and ML analyses. Leveraging the power of ML to effectively summarize mutational effects, more experimental resources can be freed up for researchers to carry out more ambitious screens to explore more amino‐acid sites and include a higher number of substitutions per site. Such expansion in both the breadth and depth of the sampling space will accelerate the discovery of useful proteins and provide insights into the structure and functional mechanisms to guide further designs.

## Conflict of Interest

A.S.L.W. would like to declare that patent applications have been filed based on their published work on the presented combinatorial genetics platforms CombiSEAL and CombiGEM‐CRISPR.
